# Splinting after contracture release for Dupuytren's contracture (SCoRD): protocol of a pragmatic, multi-centre, randomized controlled trial

**DOI:** 10.1186/1471-2474-9-62

**Published:** 2008-04-30

**Authors:** Christina Jerosch-Herold, Lee Shepstone, Adrian J Chojnowski, Debbie Larson

**Affiliations:** 1 School of Allied Health Professions, University of East Anglia, Norwich, UK; 2 School of Medicine, Health Policy and Practice, University of East Anglia, Norwich, UK; 3 Department of Trauma and Orthopaedics, Norfolk and Norwich University Hospital, Norwich, UK; 4 Occupational Therapy Department, Norfolk and Norwich University Hospital, Norwich, UK

## Abstract

**Background:**

Splinting as part of the overall post-surgical management of patients after release of Dupuytren's contracture has been widely reported, though there is variation in practice and criteria for using it. The evidence on its effectiveness is sparse, of poor quality and contradictory with studies reporting negative and positive effects.

**Methods/Design:**

A multi-centre, pragmatic, randomized, controlled trial is being conducted to evaluate the effect of static night splinting for six months on hand function, range of movement, patient satisfaction and recurrence at 1 year after fasciectomy or dermofasciectomy. Using a centrally administered computer randomization system consented patients will be allocated to one of two groups: i) splint group who will be given a static splint at approximately 10 to 14 days after surgery to be worn for 6 months at night time only as well as hand therapy; ii) non-splint group, who will receive hand therapy only. The primary outcome measure is the patient-reported Disabilities of the Arm, Hand and Shoulder Questionnaire (DASH). Secondary outcomes are total active flexion and extension of fingers, patient satisfaction and recurrence of contracture. Outcome measures will be collected prior to surgery, 3 months, 6 months and 1 year after surgery. Using the DASH as the primary outcome measure, where a difference of 15 points is considered to be a clinically important difference a total of 51 patients will be needed in each group for a power of 90%. An intention-to-treat analysis will be used.

**Discussion:**

This pragmatic randomized controlled trial will provide much needed evidence on the clinical effectiveness of post-operative night splinting in patients who have undergone fasciectomy or dermofasciectomy for Dupuytren's contracture of the hand.

**Trial Registration:**

Current Controlled Trials ISRCTN 57079614

## Background

### Dupuytren's Disease

Dupuytren's Disease (DD) is a progressive fibroproliferative disorder of the palmar fascial complex of the hand. Fascial bands form nodules and cords and eventually contract causing digital finger flexion contractures where they cross joints, especially in the metacarpophalangeal (MCP) and proximal interphalangeal (PIP) joints of the fingers. The little and ring fingers are most frequently affected. These contractures can lead to severe impairments in hand function[1]. Prevalence is highest in people of Northern European stock. The disease presents most commonly in men in their 5th decade of life and an estimated 20% of men over the age of 60 years are affected. In the UK more than 2 million people have DD many of which will require surgical intervention[1].

### Surgical and post-surgical management

Although pharmacological treatment options are being investigated[2], transection of cords (fasciotomy) or excision of diseased fascial bands (fasciectomy) with or without excision of overlying skin remain the only treatments at present [1,3]Surgery is designed to reverse digital contractures and to restore hand function vital for everyday activities of self-care, work and leisure[4]. Following surgery hand therapy provided by specialist occupational therapists or physiotherapists is seen as integral to the management of these patients and is aimed at reducing swelling, optimising wound healing, restoring finger mobility and maximising hand function[4]. In order to maintain the optimum finger extension achieved through surgical release some surgeons advocate the use of static or dynamic splints for daytime and/or night time to be worn up to 6 months. The aim of the splint is to provide prolonged stretch to healing tissues and prevent flexion contractures. Whilst the effectiveness of surgical excision is well established the benefit of different post-operative interventions including static splinting and their effect on functional outcomes remain undecided.

### Evidence on effectiveness of splinting is low quality and inconclusive

Surveys of practice among hand surgeons [5] have highlighted wide variation in the use of post-operative splints [6,7], largely due to a lack of good quality evidence to support their use.

The evidence that does exist regarding post-operative splinting is inconsistent reporting both negative [8](loss of finger flexion, delayed return to function and increased recurrence) and positive (improved scar extendibility, reduced finger extension deficits and delayed recurrence)[9] effects. Research to date is inconclusive due to the available studies being observational, small scale and heterogeneous in terms of the interventions (i.e. type of splint and wearing regimens) and assessment of outcomes.

### The need for a trial

Severe Dupuytren's contracture requiring release accounts for approximately 12,000 patients undergoing surgery in the UK per year [10]. Despite the fact that evidence on the effectiveness of post-operative interventions including splinting remains scarce it remains widely used in practice. A recent survey of 573 orthopaedic consultants in the UK showed that 33% of surgeons used splinting most or all of the time [7]. Another UK survey of 141 surgeons of various grades found that 84% advocated the use of a thermoplastic night splint, however duration of wear differed widely ranging from a few weeks to 6 months[6].

Custom-made splints are costly in terms of material and therapists' time to fabricate. For the patient they may be cumbersome and inconvenient to wear even if only at night-time, which in turn may affect adherence. Also, there is some controversy over the amount of tension [11] and the effect of tension on diseased fascia with some in vitro studies indicating that this can accelerate the fibroproliferative turnover, possibly speeding up recurrence and extension of contractures[12].

On the other hand there is also evidence that they may help in maintaining extension of digits especially where the PIP joint is involved. The rationale for using splints is that they provide continuous low-load tension to healing tissues and thus help to elongate scar tissue[11], which other modalities such as active and passive hand exercises can also achieve but which can be time-consuming for patient and therapists. In the absence of any high quality evidence to support post-operative splints their widespread use may no longer be justifiable to purchasers, providers or patients.

## Methods

### Objectives of the trial

To compare the effectiveness of post-operative static night splinting additionally to standard post-operative hand therapy in improving patient reported hand function, total active movement, patient satisfaction and recurrence at one year.

### Design

The randomized controlled trial is an accepted methodology for minimizing potential biases and ensuring high internal validity. This study is a pragmatic, multi-centre, randomized controlled trial comparing two interventions: post-operative hand therapy including a static night splint versus post-operative hand therapy without splint.

### Setting

The trial will recruit patients from five National Health Services teaching hospitals across three regions in East Anglia in the UK. All five centres have an orthopaedic service and two centres also include a plastic and reconstructive surgery service making up a total of 15 orthopaedic and plastic surgeons with an interest in hand surgery who regularly undertake fasciectomy and/or dermofasciectomy for Dupuytren's contracture (DC). Post-surgical hand rehabilitation services are provided at each centre by hand therapists (occupational therapists and/or physiotherapists with a special interest in hand injuries). Patients admitted for surgery are either operated on as day-cases or admitted overnight, depending on the extent of surgical excision and overall health status.

### Ethics

This multi-centre trial was approved by the Research Ethics Committees and Research Governance Committees for each participating hospital. Informed, written consent will be obtained from all patients prior to enrolment into the trial.

### Recruitment

Patients referred to the orthopaedic and plastic surgery out-patient clinics with a Dupuytren's contracture will be assessed and screened for eligibility by the consultant surgeons. Those meeting inclusion criteria will be told about the trial and given an information leaflet to take away with them. The waiting times between listing for surgery and actual surgery ranges from 12 to 22 weeks, therefore recruitment and consent will be collected later and closer to the date of surgery. Six to eight weeks prior to surgery patients are sent a full participant information sheet and a consent form with a self-addressed envelope by the trial co-ordinator. Those returning a signed consent form are contacted to arrange a pre-surgical baseline assessment.

### Inclusion and exclusion criteria

The target study population are patients with Dupuytren's disease affecting one or more fingers presenting at hand clinic requiring surgical release by fasciectomy or dermofasciectomy and who have consented to surgery. Exclusions are: patients under the age of 18 years, those unable to give informed consent and patients with Dupuytren's contracture affecting the first web space only.

### Randomization

Patients will be randomized into one of two groups. One group will receive post-operative hand therapy only and the other group will receive hand therapy and a static night splint to be worn for 6 months. Randomization will be stratified by recruiting hospital (5 centres) and surgical procedure (dermofasciectomy or fasciectomy) in block lengths of 4.

Patients are randomized after surgery. This will ensure operating surgeons are unaware of group allocation at the time of surgery. A telephone based central computerised randomization service will be used to generate the allocation sequence and the treating hand therapists will obtain the group allocation just before or at the first post-operative hand therapy appointment by ringing a dedicated telephone number available 24 hours. Neither the patients nor the treating hand therapists can be blinded to group allocation.

### Interventions

Surgical procedure, that is approach and extent of surgical excision as well as post-operative hand therapy, will not be standardised. Surgeons vary in their preference and skill for different procedures and it is difficult to standardise these. Immediate post-operative management including the use of plaster of Paris or thermoplastic resting splints, type of dressing and time to removal of sutures varies between surgeons and centres. Local standard protocols will be followed. For the purposes of this trial the intervention period commences at the first hand therapy appointment which is normally after suture removal (7–14 days after surgery). Hand therapy will be tailored to the patients presenting problems and needs and is aimed at reducing oedema, promoting wound healing and guiding post-surgical scarring, maximising finger range of movement and facilitating full return to functional use of the hand. This can be achieved through a wide range of modalities. The hand therapists who deliver the treatment will use a standard checklist to record the number of sessions each patient receives as well as the modalities and treatments received at each session.

#### Splint group

The use of a static night splint is standard practice in all 5 participating centres for the majority of patients, consequently the provision of the night splint in this trial does not constitute a new intervention. Therapists already have training and experience in manufacturing custom-made splints for DC and local protocols and procedures regarding choice of material and design will be followed. Splints will be static with no dynamic components. The principle of 'no tension' ([11,13]will be used. This involves placing the operated digits in maximum achievable extension without placing tension on the surgical wound. Any non-correctable contractures at the time of surgery will be accommodated in the splint.

Where splints are contra-indicated, for example due to post-surgical complications, the application of the splint is delayed. Patients allocated to the splint group will be asked to wear splints at night-time only for 6 months. They are given a simple splint diary in which they record on a weekly basis how many nights out of 7 they actually wore the splint and reasons for not doing so. The splint diary will be collected at the 3 months and 6 months follow-up visit by the research associates.

#### No splint group

Patients allocated to the no-splint group will receive hand therapy as described above only. During the early post-operative phase and especially where the surgical scar crosses the metacarpophalangeal (MCP) or interphalangeal (IP) joints patients may experience loss of extension due to the tendency of scar tissue to contract. Such a loss of finger extension may be temporary and also may respond to other treatment modalities such as active exercises, scar massage etc., however where this does not resolve within a week and exceeds an acceptable threshold patients will be given a splint. The criteria for such protocol deviations have been devised and agreed by all participating centre surgeons and therapists. The first hand therapy visit after surgery will be used to record baseline active extension at MCP and proximal interphalangeal (PIP) joints. On subsequent visits this is reassessed and if loss of extension compared to this baseline is greater than 20° at the PIP joint or greater than 30° at the MCP joint patients will be given a splint. Protocol deviations and reasons for these will be recorded.

### Outcomes

Surgical release of a Dupuytren's contracture is primarily designed to 'open up' the palm and restore finger extensibility and improve hand function. Past studies of splinting have reported outcomes in terms of total active extension and total active flexion as well as self-reported symptoms, function and disability.

The primary outcome measure is self-reported hand function and disability assessed by the Disabilities of Arm Shoulder and Hand Questionnaire (DASH)[14]. The DASH is a 30-item patient-reported questionnaire designed to measure, symptoms and physical function in patients with musculoskeletal disorders of the upper extremity. There is no disease-specific outcome instrument for Dupuytren's contracture and the validation of the DASH has been done on a wide range of patients including Dupuytren's contracture. Longitudinal construct validity has been assessed in patients including those with DC and the responsiveness assessed by effect size is moderate (ES = 0.5). It has good concurrent validity with the subscales of the SF-36 [15] and test-retest reliability has been found to be excellent (ICC = 0.96) [16].

Secondary outcomes are range of movement, patient satisfaction and recurrence. Range of movement for digits I to IV will be assessed using a Rolyan hyperextension finger goniometer and following a standardised protocol. Total active flexion (TAF) and total active extension (TAE) will be recorded by adding individual DIP, PIP and MCP joint scores. Intra-tester reliability of finger goniometry using a standardised protocol has been shown as acceptable [17] with a within-tester error of less than 5 degrees.

Patient satisfaction will be assessed at 6 and 12 months using an 11 point verbal rating scale (VRS) with the question: How satisfied are you with the overall result of the surgery now? A score of 0 means completely dissatisfied and 10 means completely satisfied.

Recurrence of contracture in the previously operated field [10] will be assessed only at 6 months and 12 months post surgery by MCP and PIP joint extension deficit.

Baseline data on socio-demographic variables (including age, gender, handedness, previous surgery and occupational status) will be collected at the pre-operative appointment together with RoM measurements and DASH questionnaire. A number of other measures will be obtained: patients allocated to the splint group will be asked to keep a splint diary to measure splint adherence. Frequency and type of treatment received by the hand therapists will be recorded.

#### Timing of outcome measures

Baseline assessment will occur at 4 to 8 weeks prior to surgery. Follow-up assessments at 3 months, 6 months and 1 year post-surgery as well as baseline assessment will be taken by the research associates employed on the trial. Patients will be visited at home in order to measure finger range of movement with a finger goniometer using a standardised protocol and collect the completed DASH Questionnaire which is posted out prior to the visit. At the 6 months and 12 months follow-up visit the verbal rating scale for satisfaction will also be administered and the presence or absence of recurrence in contracture (as distinct from scar or joint contracture) will be assessed. The patient completed splint dairy will also be collected at 3 months and 6 months and the patient asked to rate their overall adherence with splint wear as an average proportion out of 7 days.

### Blinding

Due to the nature of this study, it is not possible to blind the patient or the treating therapist to the intervention received. The primary outcome measure is the patient-rated DASH and therefore blinded assessment is not possible. Secondary outcomes will be assessed by research associates who will not be blinded to the group allocation.

### Sample size

Using the DASH [14] as the primary outcome measure, where a difference of 15 points is considered to be a clinically important difference [16] and using a between-group standard deviation of 22 points [18] a total of 51 patients would be needed in each group for a power of 90%. Allowing for a 20% loss to follow-up a total of 128 patients need to be randomized. Based on current surgery rates in the five participating centres and assuming a consent rate of 40–50% of those patients invited to take part we anticipate that the target sample size can be recruited within 16 months.

### Analysis strategies

All analyses will be conducted using an Intention-to-treat approach using all randomized participants. The primary endpoint is assumed to follow a Normal distribution. Mean scores between groups at follow-up will be analysed using a general linear model with baseline DASH scores as a covariate and hospital and type of surgery also added as independent factors (these are used to stratify the randomization).

Range of movement and satisfaction will be similarly analysed. However, as multiple digits are likely to be involved per person, a generalised estimating equation (with a Normal error term) will be used to adjust for intra-person correlations. The proportion of individuals with recurrent DD will be compared between groups and analysed using Fisher's Exact test. Subgroup analyses will be undertaken by adding an appropriate interaction term to the general linear model or general estimating equation. An interaction between group and severity and group and type of surgery will be considered, i.e. a subgroup analysis of severity and type of surgery. It is acknowledged that the statistical power of such analyses is likely to be low but as these are of secondary concern it would be inappropriate to consider a potentially very large sample size increase simply on account of these.

## Discussion

This pragmatic randomised controlled trial will provide much needed evidence on the clinical effectiveness of post-operative night splinting in patients who have undergone fasciectomy or dermofasciectomy for DC.

## Competing interests

The authors declare that they have no competing interests.

## Authors' contributions

DL and CJH conceived the original idea. LS helped with trial design and statistical analysis and AJC provided surgical expertise to the development of the protocol and funding application. All authors are joint grantholders, contributed to the manuscript and approved the final manuscript.

## Pre-publication history

The pre-publication history for this paper can be accessed here:


